# A Triple-Precursor Blend as a Topical Solution to Protect the Skin Against Environmental Damage

**DOI:** 10.3390/biology14030266

**Published:** 2025-03-05

**Authors:** Ping Gao, Xue Xiao, Zhuang Zhou, Hong Zhang, Raghupathi Subramanian, Anuchai Sinsawat, Xuelan Gu

**Affiliations:** 1 Unilever R&D Shanghai, 66 Lin Xin Road, Shanghai 202305, China; apple.gao@unilever.com (P.G.);; 2 Unilever R&D Trumbull, 55 Merritt Blvd, Trumbull, CT 06611, USA; 3 Unilever Thai Holdings Ltd., 411 Srinakarin Road, Suanluang, Bangkok 10250, Thailand

**Keywords:** precursor, oxidative stress, skin damage, barrier protection, skin living equivalent, transcriptomics

## Abstract

The epidermis acts as the body’s primary defense, relying on components like lipids, HA and GSH for skin barrier function, hydration and resistance to oxidative stress. However, limitations in the topical application of these biomolecules call for novel approaches. This study investigates the efficacy of Pro-GHL, a blend of free fatty acids, acetylglucosamine and GSH amino acid precursors (GAPs), designed to replenish skin lipids, HA and GSH through de novo biosynthesis. Using primary human keratinocytes, Pro-GHL demonstrated superior antioxidant and anti-inflammatory capacities compared to each individual component under the challenge of UVB or blue light. In 3D skin equivalent models (EpiKutis^®^), Pro-GHL enhanced skin barrier function. In addition, Pro-GHL prevented the development of pigmentation in pigmented 3D skin equivalent models (MelaKutis^®^) subjected to UVB irradiation or Benzo[a]pyrene exposure. Together, these results highlight Pro-GHL’s potential as a novel, effective and comprehensive skincare approach to fortify the skin’s defense system from within and prevent the accumulation of tissue damage in response to extrinsic stressors.

## Simple Summary

Glutathione (GSH), lipids and hyaluronic acid (HA) coordinate to strengthen skin barrier function and ensure that the skin remains resilient against environmental aggressors and pathogens. However, topical formulations containing these biomolecules have various problems. In this study, we investigated the potential applications of Pro-GHL, a blend containing precursors of GSH, lipids and HA, in supporting skin barrier health. The findings suggest that Pro-GHL offers an efficacious approach to protecting the skin against environmental stress.

## 1. Introduction

The epidermis is the outermost layer of the skin, acting as the primary barrier between the body and the external environment. This layer is crucial not only for protection against pathogens and other environmental aggressors but also to ensure the retention of important substances within the body [1]. It is composed of a variety of components that work together to maintain skin health and integrity, among which glutathione (GSH), lipids and hyaluronic acid (HA) play vital roles.

Upon exposure to pathogens and environmental aggressors, the production of free radicals and reactive oxygen species (ROS) is provoked, triggering uncontrolled cascades of reactions, eventually causing harmful oxidative damage to biological macromolecules. The skin develops a well-organized antioxidant network to protect cells against oxidative injury, among which GSH plays a central role. Studies have discovered compounds with antioxidant properties that protect the skin against UV radiation by increasing GSH levels [2,3,4]. However, the issues of stability and bioavailability limit the use of GSH [5]. Thus, the role of GSH precursors in boosting skin antioxidant capacity has gained attention. We have previously shown that glutathione amino acid precursors (GAPs), consisting of L-cystine, L-glutamine and glycine, increase skin GSH levels and boost its antioxidant defense, thereby enhancing the skin’s ability to fight against oxidative stress and maintaining barrier function [6,7].

The stratum corneum (SC), the outermost epidermal layer, consists of corneocytes (dead skin cells) and lipid-enriched intercellular domains [8]. SC lipids, primarily ceramides, free fatty acids (FFAs) and cholesterol, are delivered to the extracellular spaces of the SC through the secretion of lamellar bodies [9]. These lipids form a matrix that strengthens the skin barrier by filling in the spaces in between the corneocytes [10]. Another critical molecule to maintaining skin barrier function is HA, a naturally occurring polysaccharide found throughout the skin, with a high concentration in the epidermis [11,12]. HA is essential for moisture retention by virtue of its ability to absorb high amounts of water. This property helps keep the epidermis well-hydrated, enhancing its barrier function and appearance [12,13]. Several studies also suggest the ability of high-molecular-mass HA to protect against oxidative stress caused by environmental stressors [14,15,16,17]. Importantly, the diffusion of aqueous material through the epidermis is blocked by SC lipids. The HA-rich area inferior to this layer may obtain water from the moisture-rich dermis and the water contained therein cannot penetrate beyond the lipid-rich boundary. The maintenance of skin hydration thus critically depends on the collaboration of epidermal lipids and HA [18].

Despite their significance, the efficacy of the direct topical application of large lipid molecules like ceramides with the aim of providing barrier benefits is still controversial and may be related to the amount of endogenous ceramide [19,20]. Although commonly used in skincare products, a lot of ceramides in skincare formulations are synthetically made and thus only natural mimics. Furthermore, many products contain only ceramides but no cholesterol or fatty acids, which may bring a lipid imbalance. The efficacy of HA-containing formulations also faces problems since it largely depends on the HA’s molecular weight [21,22]. To address these challenges, research has focused on the precursors of these essential components, free fatty acids (FFAs) and N-acetylglucosamine (NAG), acting as key building blocks for the synthesis of long-chain fatty acids and ceramides in the skin. FFAs produced by *C. acnes* induce lipid synthesis and accumulation in keratinocytes, enhancing barrier function [23]. Importantly, previous research has shown that the skin can create new longer chain fatty acids and ceramides from topically applied (C16-C18) fatty acid-containing moisturizers and cleansers to help replenish the skin’s barrier and boost SC regeneration [24,25,26]. HA is a high-molecular-weight glycosaminoglycan polymer composed of repeating units of glucuronic acid and NAG [27]. NAG has been found to increase HA production in cultured human epidermal keratinocytes [28] as well as dermal fibroblasts [29]. By boosting the skin’s natural production of longer chain fatty acids, ceramides and HA, topically applied FFAs and NAG nourish the skin from within by supporting natural barrier function and promoting skin hydration.

Importantly, lipids and HA are particularly susceptible to oxidative stress, leading to peroxidation products like 4-hydroxy-2-nonenal or malonaldehyde and low-molecular-weight HA fragments, which further induce protein damage, apoptosis, or the release of pro-inflammatory mediators [30,31]. GSH, if present, will neutralize free radicals, thereby preserving HA’s integrity and preventing lipid peroxidation.

**Figure 1:**
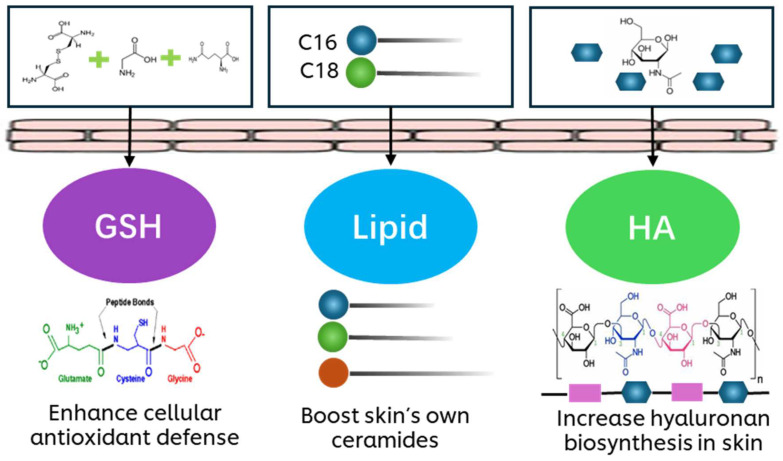
Topical precursor approach for skin protection.

In summary, lipids, HA and GSH form a multifaceted defense system that keeps the epidermis healthy, hydrated and capable of withstanding various environmental aggressors. Considering the interdependence of these components, each targeting different aspects of supporting epidermal health, a topical precursor approach combining them all would provide the most comprehensive enhancement of overall skin health. Therefore, we propose that providing a blend of three precursors (Figure 1) would be more effective than using each precursor individually. Herein, the antioxidant and anti-inflammatory efficacy of the tri-precursor blend compared to each individual component is demonstrated in primary human keratinocytes and its efficacy in skin barrier protection is further demonstrated in 3D skin equivalent models.

## 2. Materials and Methods

### 2.1. UVB-Challenged Human Keratinocytes

Primary normal human epidermal keratinocytes (NHEKs) (purchased from PromoCell, Lot: 0012602.2, Heidelberg, Germany) were maintained in EpiLife^®^ medium (ThermoFisher Scientific, Cat: M-Epi-500CF, Waltham, MA, USA) containing 60 µM calcium and 1% Human Keratinocyte Growth Supplement (HKGS) (ThermoFisher Scientific, Cat: S0015, Waltham, MA, USA) in a 37 °C 5% CO_2_ incubator. The medium was refreshed every other day. Cells before passage 6 were trypsinized at 90% confluency and used for cell viability, anti-oxidation and anti-inflammation tests.

For cell viability assessment, the cells were taken out and equilibrated at room temperature for 30 min after UVB challenge. Then, 100 μL of CellTiter-Glo^®^ Luminescent Cell Viability Assay reagent (Promega, Cat: G7571, Madison, WI, USA) was added to each well. After incubation at room temperature for 10 min to stabilize the luminescent signal, cell viability was analyzed using a Microplate Reader (Tecan, Safire2, Zurich, Switzerland).

For ROS measurement, the cells were seeded in 96-well black-walled plates at a density of 10,000 per well. The following day, GAPs (0.0001%; preparation method as reported previously), acetyl glucosamine (Pro-HA, 0.0001%; Bloomage, Jinan, Shandong, China), triple-pressed stearic acid (Pro-Lipid, 0.001%; Palm-Oleo, Klang City, Malaysia), or the tri-precursor blend (Pro-GHL, 0.0001% GAP +0.0001% Pro-HA+ 0.001% Pro-Lipid) was added into the culture medium and the cells were incubated for 24 h. After incubation, the cells were challenged with 200 mJ/cm^2^ UVB (Analytik Jena GmbH, Ultraviolet Crosslinker CL-1000, Upland, CA, USA). After UVB challenge, the cells were incubated with the culture medium containing the CellRox^®^ Green Reagent (ThermoFisher Scientific, Cat: C10444, Waltham, MA, USA) for 30 min at 37 °C. After incubation, the culture medium was removed and the cells were washed three times with PBS. Then, ROS production was measured using the Microplate Reader with excitation/emission wavelengths at 485/520 nm. All data were normalized by cell viability.

For the anti-inflammation test, the cells were treated with GAPs, acetyl glucosamine, triple-pressed stearic acid, or the tri-precursor blend as previously described and then challenged with 200 mJ/cm^2^ UVB. IL-8 was measured 24 h post UVB challenge using the Human IL-8 ELISA Set (BD Biosciences, Cat: 3223421, Franklin Lakes, CA, USA) according to the manufacturer’s instructions. All data were normalized by cell viability.

### 2.2. Oxidative Stress-Challenged Human Keratinocytes

Primary NHEKs (purchased from Guangdong Biocell Biotechnology Co., Ltd., Lot: Ep23082401, Guangzhou, China) were seeded in a 24-well cell culture plate and maintained in a 37 °C 5% CO_2_ incubator until the confluency reached 40–60%. Then, the cells were treated with Pro-GHL at different concentrations as follows: 0.0001% (0.0001% GAP +0.0001% Pro-HA+ 0.001% Pro-Lipid), 0.0002% (0.0002% GAP +0.0002% Pro-HA+ 0.002% Pro-Lipid) and 0.0005% (0.0005% GAP +0.0005% Pro-HA+ 0.005% Pro-Lipid). In the other groups, the cells were maintained in plain culture medium. In this test, blue light was employed as an oxidative stress inducer. After incubation for 24 h, the blue light and Pro-GHL groups were exposed to 10 J/cm^2^ blue light (Chengyue, Cat: cy-bz 120I, Guangzhou, China). After blue light exposure, the DCFH-DA probe (Beyotime Biotechnology, Cat: S0033M, Shanghai, China) was added into the cell culture medium and the cells were incubated at 37 °C for another 30 min. After incubation, the cell culture medium containing the DCFH-DA probe was discarded and the cells were washed with PBS three times. Then, the ROS levels in the cells were quantified using Image Pro Plus (Media Cybernetics Inc., Rockville, MD, USA) from the images acquired using a microscope (Olympus, BX43, Center Valley, PA, USA).

### 2.3. Transcriptomic Profiling of Blue Light-Challenged Human Keratinocytes Using RNA Sequencing

Primary NHEKs (purchased from Guangdong Biocell Biotechnology Co., Ltd, Lot: Ep23082401, Guangzhou, China) were used. In the non-treated (NT) group, the cells were incubated in plain culture medium. In the blue light group, the cells were incubated in plain cell culture medium and then exposed to 10 J/cm^2^ blue light. In the Pro-GHL group, the cells were pretreated with Pro-GHL (0.0001%) for 24 h followed by 10 J/cm^2^ blue light exposure. All samples were collected 24 h post blue light irradiation for RNA sequencing. RNA concentration was measured using a NanoDrop 2000 (Thermo Fisher Scientific, Waltham, MA, USA) and RNA quality was determined using an Agilent 4200 (Agilent Technology, Santa Clara, CA, USA). RNA sequencing was conducted using an Illumina NovaSeq 6000 by Berry Genomics (Beijing, China).

Clean reads were mapped to the human genome (GRCh38) using Hisat2. Differential expression analysis was conducted using edgeR. Differentially expressed genes (DEGs) were identified with cut-offs of *p*-value < 0.05 and fold change > 1.5. GO enrichment analysis was performed using clusterProfiler (R version 4.4.1) and pathway analysis was performed using Ingenuity Pathway Analysis (IPA, QIAGEN, Hilden, Germany).

### 2.4. Application of the LSE Model and Histological Analysis

Three-dimensional skin equivalent models (LSE, purchased from Guangdong Biocell Biotechnology Co., Ltd, EpiKutis^®^, Lot: ES240703, Guangzhou, China) were utilized to analyze the histological changes. In the NT group, only the culture medium was refreshed daily. In the Pro-GHL group, the models were treated with 0.001% Pro-GHL for five days with daily culture medium refreshment. After the last application, the EpiKutis^®^ models were cultured for another 24 h and collected for histological analysis.

The collected models were immediately fixed in cold 4% neutral buffered formalin solution (SIGMA-ALDRICH, Co., Cat. 252549, Saint Louis, MO, USA), dehydrated and embedded in paraffin. Then, the tissues were sliced into 5 μm vertical sections. The sections were further deparaffinized and rehydrated through graded ethanol series and stained with hematoxylin and eosin (H&E) (Beyotime Biotechnology, Cat: C0107 and C01055, Shanghai, China) for histological evaluation. The boundary of the living cell in the epidermal layer was drawn and the average distance between the boundaries was considered as the epidermal living cell thickness and this value was calculated using Image Pro Plus.

### 2.5. Application of the pLSE Model and Measurement of L* and Melanin Content

UV- and Benzo[a]pyrene (BaP)-challenged pigmented 3D skin equivalent models (pLSE, purchased from Guangdong Biocell Biotechnology Co., Ltd., MelaKutis^®^, MS240801, Guangdong, China) were used to evaluate the protective effects of Pro-GHL. In the UV challenge study, the Pro-GHL group was irradiated with 50 mJ/cm^2^ UVB and treated with 0.0001% Pro-GHL for six days. Only the culture medium was refreshed daily in the NT group. The UV group was only exposed to 50 mJ/cm^2^ UVB daily. In the BaP challenge study, the Pro-GHL group was treated with fresh medium containing 3 μM BaP (SIGMA-ALDRICH, Co., Cat: B1760, Saint Louis, MO, USA) and 0.0001% Pro-GHL for six days with daily culture medium refreshment. The BaP group was treated with only BaP and the NT group was maintained in daily refreshed plain culture medium for six days. After the last application, the MelaKutis^®^ models were cultured for an additional 24 h. Then, the models and culture medium were collected for further analysis.

L* value is a well-known measurement of the brightness of skin color. To measure the L* value, the MelaKutis^®^ models were placed on a flat and hard white plane with the cuticle upward and aligned in the detection hole of the Colormeter (Cortex Technology, DSM II, Nordjylland, Hadsund, Denmark). Then, the L* value of each model was recorded three times from the readings of the Colormeter.

After measuring the L* value, three MelaKutis^®^ models from each group were used for melanin content analysis. The models were rinsed with 1 mL PBS buffer followed by a mixture of ddH_2_O with ethanol and ether. Then, the model was lysed in 1 mL 1 M NaOH containing 10% DMSO and incubated in a 80 °C water bath for 40 min. After incubation, 200 μL of the supernatant was transferred to a 96-well plate and the absorbance value was obtained at 405 nm to measure the melanin content.

MelaKutis^®^ models from each group were also reserved for histological analysis. The tissue sections were prepared as described in Section 2.4.

### 2.6. RNA Extraction and Real-Time Quantitative Polymerase Chain Reaction (qPCR)

Total RNA was extracted from the MelaKutis^®^ samples for real-time qPCR analysis. After application, the MelaKutis^®^ samples were rinsed with PBS and RNAiso Plus (Accurate Biotechnology, AG21102, Changsha, China) was added to lyse the samples. Then, total RNA was extracted with chloroform. The extracted RNA was quantified using a Nanodrop spectrometer (Thermo Fisher Scientific, Rockford, MA, USA) and reverse transcribed to generate the template cDNA using the Evo M-MLV RT Premix for qPCR (Accurate Biotechnology, Cat: AG11706, Changsha, China) according to the manufacturer’s protocol. The expression levels of ELOVL1 (F: 5′-AGGCTTATGTGGTCAGGACTG-3′; R:5′-TGGAGTGGCAGACAGCAAT-3′), CD44 (F:5′-GGAACAGTGGTTTGGCAACAGA-3′; R:5′-TGCATTGGATGGCTGGTATGA-3′) and HMOX1 (F:5′-TTGCCAGTGCCACCAAGTTC-3′; R:5′-TCAGCAGCTCCTGCAACTCC-3′) were analyzed via qPCR using a SYBR^®^ Green Premix Pro Taq HS qPCR Kit (Accurate Biotechnology, AG11701, Changsha, China). The actin beta (*ACTB*) gene was selected as the housekeeping gene and all data of the target genes were normalized to ACTB expression levels. The fold changes in expression were calculated relative to the NT group.

### 2.7. Immunohistochemical (IHC) Staining

Sections from EpiKutis^®^ and MelaKutis^®^ were reserved for IHC staining. IHC analyses for Hyaluronan Binding Protein (HABP) and loricrin were prepared with the primary antibodies against HABP (AMSBIO, Cat: AMS.HKD-BC41, Cambridge, MA, USA) and loricrin (Abcam, Cat: ab198994, Waltham, MA, USA) according to the manufacturer’s instructions. The sections were finally incubated with a DAB chromogen and dehydrated before being cover-slipped. Then, the slides were examined under a microscope (Leica, DM2500, Wetzlar, Hessen, Germany). All of the staining images were quantified with Image Pro Plus in triplicate. The staining procedure for melanin was similar. Briefly, the melanin distribution was assessed with a Masson–Fontana melanin staining kit (Yike Biotechnology Service Co., Ltd, Cat: YK2318, Xi’an, China) according to the manufacturer’s instructions.

### 2.8. Statistical Analysis

The results of the in vitro tests are presented as the mean ± standard deviation (SD) in the figures. The statistical significance was determined using Student’s *t*-tests with two tails and equal SD. *p* < 0.05 was considered statistically significant.

## 3. Results

### 3.1. The Effects of GAPs, Pro-HA, Pro-Lipid and Pro-GHL in UVB-Exposed Keratinocytes

**Figure 2:**
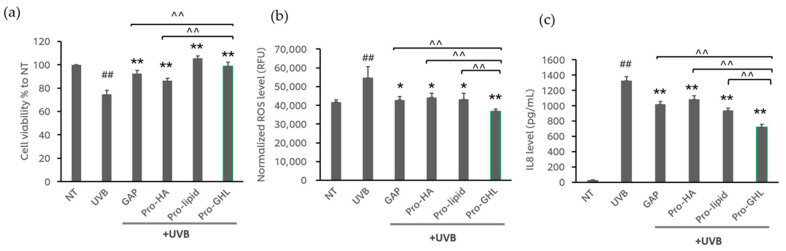
Pro-GHL suppressed ROS and IL-8 levels in UVB-exposed keratinocytes. (**a**) Cell viability of UVB-exposed NHEKs treated with Pro-GHL and individual components. (**b**) ROS level in UVB-exposed NHEKs treated with Pro-GHL and individual components. (**c**) Relative normalized level of IL-8 in UVB-exposed NHEKs. All values are the mean ± SD (n = 3). ## *p* < 0.01 versus the NT group; * *p* < 0.05 versus the UVB group; ** *p* < 0.01 versus the UVB group; ^^ *p* < 0.01 versus the Pro-GHL group.

ROS and IL-8 levels were measured in the control and UVB irradiated keratinocytes with and without Pro-GHL with each component at 0.0001% *w*/*w*. As shown in Figure 2a, UVB exposure reduced cell viability to 74.9% compared to the NT group. Pre-application of GAPs, Pro-HA, Pro-Lipid and Pro-GHL increased cell viability to 92.6%, 86.6%, 105.6% and 99.1%, respectively. Considering the fact that the differences could be caused by cell viability, ROS production and IL-8 secretion were normalized to cell viability. As shown in Figure 2b,c, as expected, UVB exposure significantly induced ROS production and IL-8 release. Pre-application of GAPs, Pro-HA, Pro-Lipid, or Pro-GHL significantly inhibited ROS production as compared to UVB-treated cells. Among all of the applications, Pro-GHL showed a greater reduction in ROS level (32.2% inhibition compared to the UVB group, *p*-value < 0.01) compared to each individual component in Pro-GHL (Figure 2b). Furthermore, the elevated IL-8 level triggered by UVB was suppressed by Pro-GHL (48.5% reduction, *p*-value < 0.01, compared to the UVB group), GAPs (18.4% reduction, *p*-value < 0.01, compared to the UVB group), Pro-HA (29.3% reduction, *p*-value < 0.01, compared to the UVB group) and Pro-Lipid (40.5% reduction, *p*-value < 0.01, compared to the UVB group). Notably, Pro-GHL exhibited a greater reduction in IL-8 compared to the other groups. These results suggest that the combination of the three precursor components leads to a synergistic effect, enhancing the antioxidant capacity and strengthening the anti-inflammatory activity of each component for better protection of the skin under UVB exposure.

### 3.2. Dose–Response Effect of Pro-GHL on Blue Light-Exposed Keratinocytes

**Figure 3:**
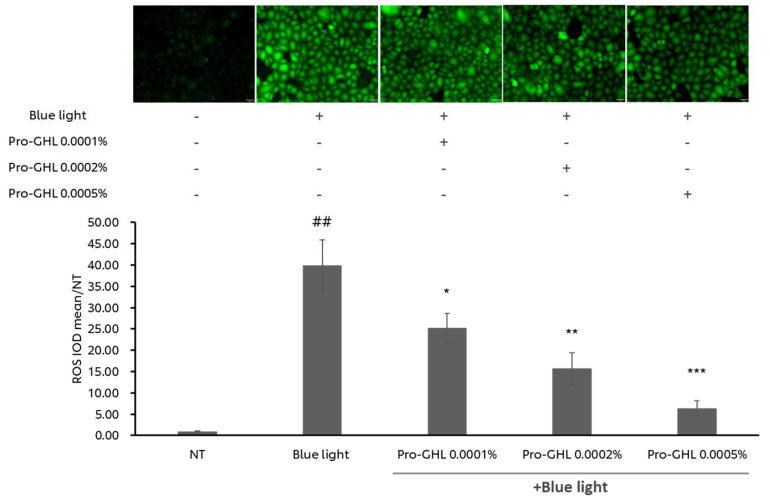
Dosage–responsiveness of ROS inhibition by Pro-GHL in NHEKs. NHEKs treated with or without Pro-GHL were exposed to blue light. Levels of ROS in NHEKs were quantified using the DCFH-DA probe. All values are the mean ± SD (n = 3). ## *p* < 0.01 versus the NT group; * *p* < 0.05 versus the blue light group; ** *p* < 0.01 versus the blue light group; *** *p* < 0.001 versus the blue light group.

Blue light is a well-known inducer of oxidative stress. To demonstrate the anti-oxidative capabilities of Pro-GHL, ROS was analyzed in blue light irritated keratinocytes. As shown in Figure 3, blue light exposure dramatically stimulated ROS production in the keratinocytes by around 39 times. Pretreating the cells with 0.0001%, 0.0002%, or 0.0005% Pro-GHL significantly suppressed ROS generation in a dose-dependent manner. This result indicates that Pro-GHL was a strong antioxidant against blue light-induced oxidative stress.

### 3.3. Transcriptomic Analysis of Pro-GHL in Blue Light-Challenged Keratinocytes

To better understand the effect of the combination of the three precursors on the ROS-challenged keratinocytes, blue light-exposed NHEKs were treated with Pro-GHL to investigate the gene expression perturbation and biological processes involved.

**Figure 4:**
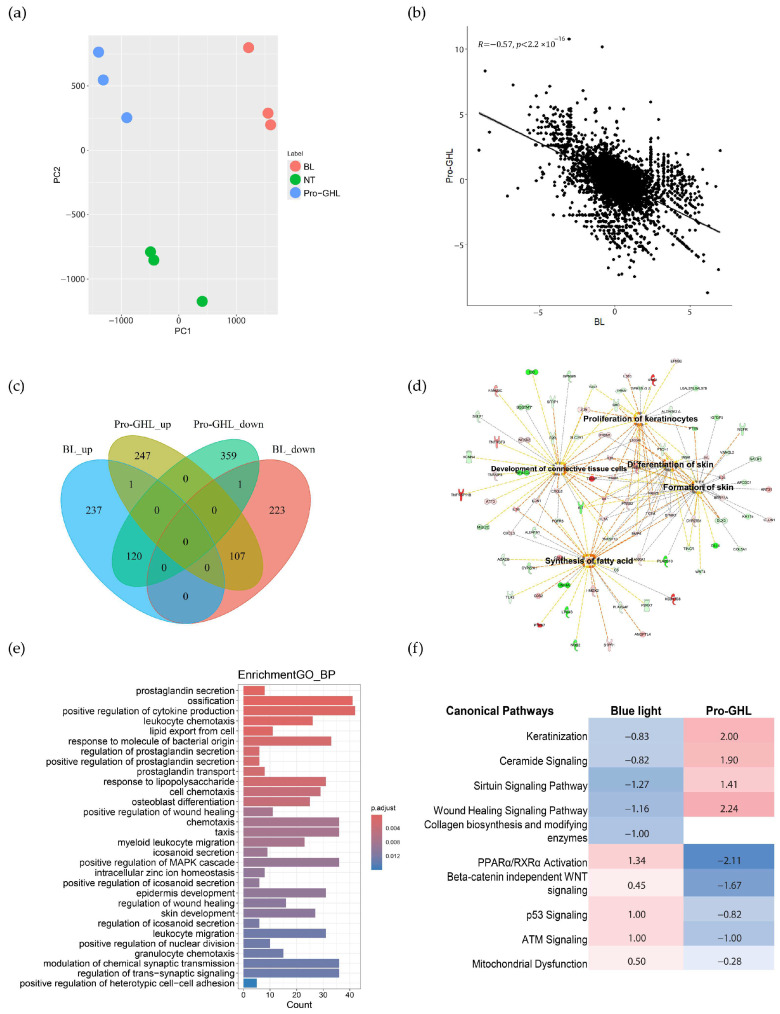
Pro-GHL restored gene expression changes induced by blue light and regulated multiple pathways linked to the skin barrier. (**a**) Principal component analysis of gene expression in the Pro-GHL, blue light and control groups (BL: blue light group; NT: control group). (**b**) Correlation between gene expression changes in the Pro-GHL and blue light groups. (**c**) Venn diagram of DEGs (BL_up: upregulated DEGs in the blue light group versus the control group. BL_down: downregulated DEGs in the blue light group versus the control group. Pro-GHL_up: upregulated DEGs in the Pro-GHL group versus the blue light group. Pro-GHL_down: downregulated DEGs in the Pro-GHL group versus the blue light group). (**d**) Highlighted functions of Pro-GHL-regulated genes predicted via IPA (Green: downregulation. Red: upregulation. Orange: predicted activation of genes based on Pro-GHL-regulated DEGs). (**e**) Top enriched biological processes of DEGs in the Pro-GHL group based on GO enrichment analysis. (**f**) Comparison of IPA-predicted pathway activity between the Pro-GHL and blue light groups. The number indicates the Z-score of the pathway from the IPA where a positive number indicates activation of the pathway and a negative number indicates inhibition of the pathway.

Whole-transcriptome gene expression profiling demonstrated the protective effect of Pro-GHL against blue light. Firstly, the principal component analysis showed that there were clearly three groups (Figure 4a) and the gene expression change between Pro-GHL and blue light challenge had a negative coefficient (Figure 4b). Secondly, the common DEGs between the Pro-GHL application group and the blue light challenge group illustrated that almost all common DEGs showed opposing directions of regulation, indicating restoration by Pro-GHL (Figure 4c).

The dysregulated DEGs induced by blue light were involved in biological processes including keratinization, lipid transport and pathways including lipid metabolism, extracellular matrix organization, circadian rhythm signaling, interferon gamma signaling and tight junction signaling. Pro-GHL-regulated genes were engaged in multiple skin-relevant biological processes and pathways such as lipid export, skin development, keratinocyte differentiation, epidermal development and wound healing regulation. Skin benefit-related functions and relevant genes were revealed using IPA analysis and the network was highlighted (Figure 4d). More biological processes enriched by Pro-GHL are shown in Figure 4e. At the level of individual genes, Pro-GHL upregulated genes linked to the skin barrier, such as *GRHL1*, *GRHL3*, *IVL*, *ZNF750*, *OCLN*, *OVOL1*, *TJP1* and *CLDN1*. The pathway comparison demonstrated that the activity score of common pathways predicted using IPA in the Pro-GHL and blue light groups was negative, consolidating the protective effect of Pro-GHL, especially the protective effect through the regulation of keratinization, lipids, ceramide signaling and mitochondrial dysfunction (Figure 4f).

### 3.4. The Effects of Pro-GHL on the 3D Skin Equivalent Model

**Figure 5:**
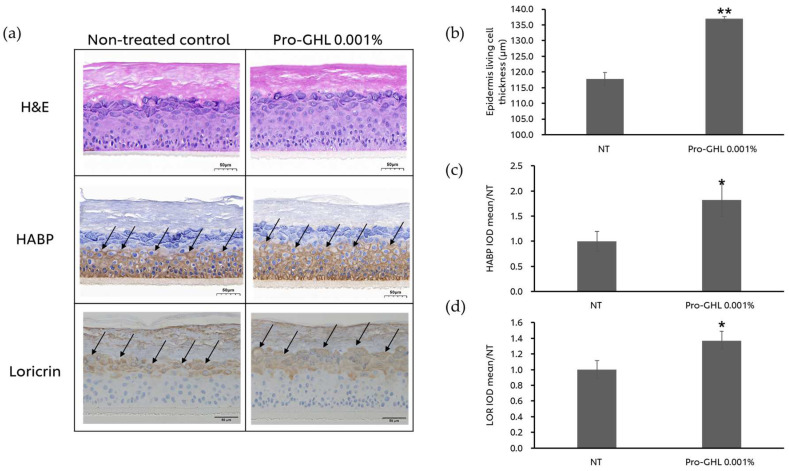
The effects of Pro-GHL on the skin barrier. (**a**) H&E, IHC of HABP and loricrin staining of the 3D skin equivalent model. The scale bar equals 50 μm. Arrows indicate IHC labeling of HABP (brown) or loricrin (brown). (**b**–**d**) Quantification of epidermal living cell thickness, HABP and loricrin levels. All values are the mean ± SD (n = 3). * *p* < 0.05 versus the NT group; ** *p* < 0.01 versus the NT group.

To validate the effect of Pro-GHL on the skin barrier, a 3D skin equivalent model was applied. After treating EpiKutis^®^ with 0.001% Pro-GHL, epidermis thickness, HABP and loricrin were analyzed. The results (Figure 5) illustrated that Pro-GHL increased the thickness of living cells by 16.25%, along with elevation of HABP and loricrin expression by 81.90% and 36.91%, respectively. These results indicated that Pro-GHL promoted skin barrier differentiation and hydration, resulting in a strengthened skin epidermis.

### 3.5. The Effects of Pro-GHL on the UV- and Pollutant-Challenged Pigmented 3D Skin Equivalent Model

**Figure 6:**
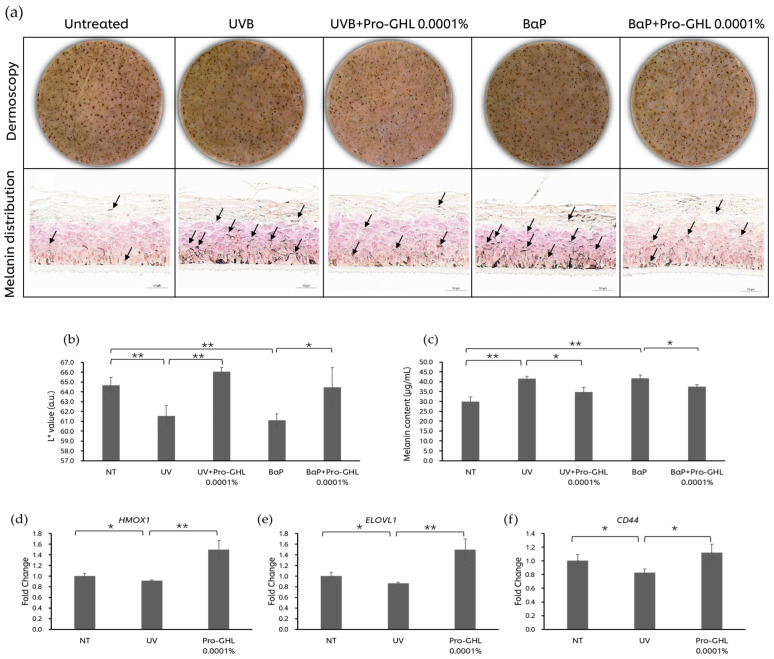
Protective effects of Pro-GHL against UV and BaP. (**a**) Dermoscopy and melanin distribution of the pigmented 3D skin equivalent model. The scale bar equals 50 μm. Arrows indicate the melanin pigment. (**b**,**c**): L* value and total melanin content of the pigmented 3D skin equivalent model after application. (**d**–**f**) Gene expression of *HMOX1, ELOVL1* and *CD44* in the pigmented 3D skin equivalent model under UVB with or without Pro-GHL application. All values are the mean ± SD (n = 3). * *p* < 0.05 between groups; ** *p* < 0.01 between groups.

L* value is a well-known indicator of overall tone. After UVB exposure and BaP application, the L* value of the MelaKutis^®^ significantly declined by 4.81% and 5.50%, respectively (Figure 6). Of note, 0.0001% Pro-GHL application reversed the L* value decline induced from UVB and BaP by 7.31% and 5.45%, respectively. These results were consistent with the melanin content level of the MelaKutis^®^ and the melanin distribution in the sample sections (Figure 6). UVB and BaP stimulated melanin production by 38.76% and 39.42% as compared with the untreated group. And 0.0001% Pro-GHL reduced this level by 16.29% and 10.44%, respectively.

The results of the qPCR in the MelaKutis^®^ showed that UV exposure reduced *HMOX1*, *ELOVL1,* and *CD44* levels, indicating that UVB affected the anti-oxidative capability, lipid synthesis and barrier function of the skin. After application of Pro-GHL, the gene expression levels of *HMOX1*, *ELOVL1* and *CD44* significantly increased (Figure 6).

## 4. Discussion

Skin, as the body’s largest organ, serves as the foremost barrier between our internal physiology and the external environment. Lipids like ceramides, cholesterol and fatty acids form a “mortar” that holds skin corneocytes together, maintaining hydration and integrity [32]. HA, a natural moisturizer, helps to retain water in the skin, enhancing its plumpness and elasticity. GSH is the most prominent non-protein antioxidant, effectively protecting the skin from oxidative stresses. Together, these components ensure that the skin remains resilient against irritants, pollutants and pathogens while keeping it hydrated and healthy. Nonetheless, topically applied ceramides or HA do not penetrate effectively and are not naturally produced and are therefore not as efficient at replenishing the skin barrier. It has been previously shown in separate studies that providing skin-natural fatty acids, NAG and GAPs as precursors promotes the synthesis of longer chain fatty acids, HA and GSH, respectively [7,26,28,29]. Lipids, HA and GSH coordinate to strengthen barrier function and ensure that the skin remains resilient. Therefore, exploring the potential efficacy of the combination of these three components for improving barrier health is of interest. Formulations combining amino acids and antioxidants with HA or fatty acids have displayed comprehensive benefits and become a growing trend in recent years [33,34]. However, the integration of ingredients that stimulate the endogenous production of lipids, HA and GSH simultaneously is described, to our knowledge, for the first time herein. In this study, we provide in vitro evidence from different models supporting the skin protection capacity of Pro-GHL comprising GAPs as Pro-GSH, C16 and C18 as Pro-Lipid and NAG as Pro-HA. Our data demonstrate the following: (1) Pro-GHL showed greater potential than individual components to provide protection against inflammation and ROS induced by UVB in keratinocytes; (2) Pro-GHL inhibited ROS in a dose-response manner in blue light-challenged keratinocytes; (3) Pro-GHL strengthened skin barrier function in the LSE model; (4) Pro-GHL prevented the development of pigmentation in the pLSE model under UVB and BaP challenges; (5) Pro-GHL upregulated the expression levels of *ELOVL1*, *CD44,* and *HMOX1* in the UVB-challenged pLSE model; and (6) Pro-GHL-containing lotion exhibited skin brightening benefits in the UVB-challenged pLSE model.

Mounting evidence has demonstrated that ROS are responsible for UVB-induced skin damage [35,36]. UVB-induced ROS is subsequently capable of activating the NF-κB signaling pathway, one of the most important signaling pathways in the process of skin inflammation and aging. Activated NF-κB subsequently promotes the release of various proinflammatory cytokines and chemokines [37], leading to compromised barrier function [38]. For example, IL-8, assessed herein, is a potent chemotactic and proinflammatory cytokine that can negatively affect the skin barrier [39]. Herein, we show that in keratinocytes, Pro-GHL has a stronger antioxidant and anti-inflammatory effects in inhibiting ROS and IL-8 induced by UVB radiation than Pro-HA, Pro-Lipid, or GAPs alone (Figure 2).

Additional studies using epidermal keratinocytes as a model exposed to blue light, which is a widely used inducer of oxidative stress, demonstrated dose-dependent suppression of ROS induction by Pro-GHL (Figure 3). This also supports the hypothesis that combining GSH with skin structural biomolecules like lipids and HA may aid in its neutralization of extrinsically induced ROS. Mechanistically, RNA sequencing analysis revealed that in keratinocytes challenged by blue light, Pro-GHL pre-application altered the expression of genes associated with epidermal development, keratinocyte differentiation, ceramide signaling and wound healing (Figure 4). These mRNA responses corresponded well with the biological functions of lipid and HA precursors in the composition of Pro-GHL, which play key roles in the correct establishment of a functional barrier [40,41].

Furthermore, the LSE model was treated with Pro-GHL and the epidermal thickness evidently increased (Figure 5). Thicker epidermal layers allow for reduced water loss and better control of molecules moving across the skin through longer diffusion paths and the epidermis tends to become thinner during aging or under sunlight exposure. FFAs in the Pro-GHL may have promoted the formation of a denser and more intact lipid matrix, which contributes to epidermal integrity. In addition, Pro-GHL increased the expression of key epidermal structural proteins such as loricrin and HABP that are used like antibodies to detect HA [42] (Figure 5). Loricrin is a cysteine-rich cornified cell envelope (CE) protein whose expression is redox-sensitive and may be promoted by the epidermis-intrinsic redox homeostasis maintained by the GSH system. These results suggest the benefit of Pro-GHL in enhancing skin barrier function through the upregulation of epidermal CE components and HA.

Based on the above insights on the function and mechanism of Pro-GHL, we asked whether Pro-GHL could be a candidate to protect against skin damage caused by environmental aggressors, for instance, hyperpigmentation. In the pLSE model, Pro-GHL significantly counteracted skin darkening caused by UVB and BaP, as demonstrated by decreased melanin content and increased L* (Figure 6). Additionally, we also topically applied a Pro-GHL-containing facial serum on this model under the same conditions and the brightness was similarly improved (Figure S1). Oxidative stress and inflammation are major causal factors of melanogenesis under environmental stressors [43,44,45]. The findings in the pLSE model, along with the inhibitory effect on ROS and IL-8 in keratinocytes, prompted us to conclude that Pro-GHL could elevate the intracellular defense system through ROS inhibition and anti-inflammation against UV and pollutants in the epidermal keratinocytes, thus suppressing melanogenesis in melanocytes and delivering skin-brightening benefits.

Exposure to UVB results in pigmentation in a dynamic and non-linear way, with an acute phase and an adaptation phase, after which the skin becomes obviously pigmented [46]. We thus believe that applying Pro-GHL early after UV exposure would be a promising intervention approach. In the pLSE model, Pro-GHL was cotreated with UVB from the first exposure and the expression levels of *CD44*, *ELOVL1* and *HMOX1* were evaluated. *CD44* is the adhesion receptor to HA; *ELOLV1* encodes a key enzyme in the elongation of fatty acids; and *HMOX1* encodes Heme Oxygenase 1 (HO-1), which is a primary antioxidant and cytoprotective enzyme. The expression of these genes was decreased by UVB and increased by Pro-GHL (Figure 6), suggesting the efficacy of the three precursors correspondingly and that Pro-GHL could mitigate the molecular changes underlying UVB-induced damage, preventing subsequent morphological changes.

Our previous findings indicate that GAPs may counteract UVB-induced skin aging, as evidenced by repressing age-related inflammatory signaling pathways [6]. In line with this, our study in keratinocytes showed that pre-application with Pro-GHL inhibited UVB-induced ROS and inflammatory cytokines and reversed the impact on collagen biosynthesis, antioxidant response and apoptosis of blue light exposure. At the molecular level, this could be achieved via upregulation of the sirtuin pathway and downregulation of the p53 pathway (Figure 4). Given that HA homeostasis is involved in both intrinsic and extrinsic skin aging [47] and HA-based formulations have been shown to be effective in treating skin aging [48], to explore the potential applications of Pro-GHL in anti-inflammaging and photoaging protection would be another promising future direction.

This study has potential limitations. Although we used multiple models, the protective effect of Pro-GHL was evaluated only for cellular changes in vitro. A separate clinical study to investigate the superior effects of Pro-GHL compared to its individual component in vivo, with an emphasis on phenotype observation, would be desirable in the future. Moreover, our RNA-seq results revealed potential regulation of skin immune function by Pro-GHL. The skin is an immune organ that can produce its own antibodies that fight off infections [49]. Future work using more complex immune-competent skin models is needed to explore whether Pro-GHL could help strengthen local immunity.

## 5. Conclusions

In conclusion, the present study suggests that the combination of skin equivalent precursors is a novel and effective strategy to improve the skin’s defense system from within and prevent the accumulation of tissue damage in response to extrinsic stressors by replenishing lipids, HA and GSH through de novo biosynthesis.

## Data Availability

The data of this study are available from the corresponding author upon reasonable request with the permission of Unilever.

## Supplementary Materials

The following supporting information can be downloaded at: https://www.mdpi.com/article/10.3390/biology14030266/s1. Figure S1: UV protection of Pro-GHL-containing facial serum.
