# Supplementary material for: A Triple-Precursor Blend as a Topical Solution to Protect the Skin Against Environmental Damage
Source: Biology (Basel). 2025 Mar 5;14(3):266. doi: 10.3390/biology14030266 (PMC11939934; doi:10.3390/biology14030266)
Supplement: Supplementary file 1 [file biology-14-00266-s001.zip › biology-3458605-supplementary.pdf]

Materials and Methods

Pigmented 3D skin equivalent (MelaKutis®, MS240801, Biocell Biotech, Guangzhou, China) models were challenged with daily UVB irradiation (50 mJ/cm^2). The Pro-GHL-containing facial serum at a dose of 2mg/cm^2 was applied topically on day 2, day 4, and day 6. After the last application, all MelaKutis® models were collected for further analysis. L* values were measured using a Chroma Meter (Denmark, DSM II), and the readout was recorded three times. The models were rinsed with 1 mL PBS buffer followed by a mixture of ddH2O with ethanol and ether. Then, the model was lysed in 1 mL 1 M NaOH containing 10% DMSO and incubated in an 80°C water bath for 40 minutes. After incubation, 200 μL of the supernatant was applied to a 96-well plate, and the absorption value of the liquid was obtained at OD405 to measure the melanin content.

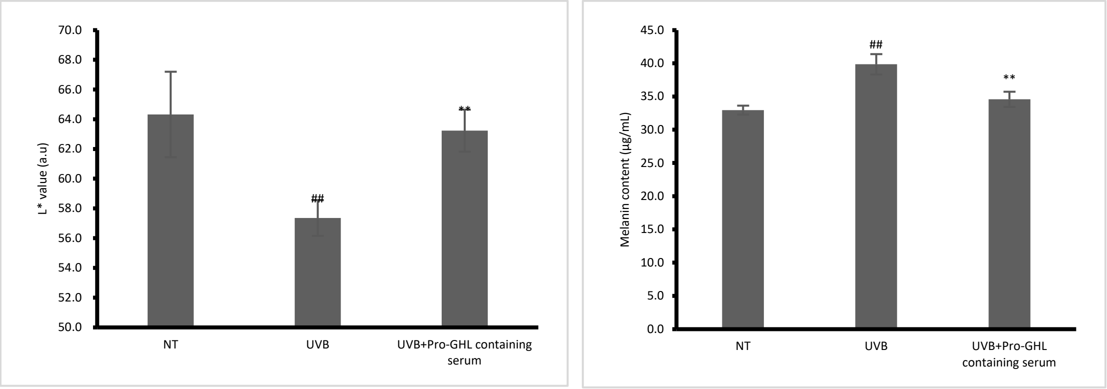

Figure S1. UV protection of Pro-GHL-containing facial serum. (a) L* value of the pigmented 3D skin equivalent model. (b) Total melanin content of the pigmented 3D skin equivalent model. All values are the mean±SD (n=3). *: $p < 0.05$ between groups; **: $p < 0.01$: between groups.
